# Lessons from the frontline: The COVID-19 pandemic emergency care experience from a human resource perspective in the Pacific region

**DOI:** 10.1016/j.lanwpc.2022.100514

**Published:** 2022-07-05

**Authors:** Claire E. Brolan, Sarah Körver, Georgina Phillips, Deepak Sharma, Lisa-Maree Herron, Gerard O'Reilly, Rob Mitchell, Mangu Kendino, Penisimani Poloniati, Berlin Kafoa, Megan Cox

**Affiliations:** a School of Public Health, Faculty of Medicine, The University of Queensland, Brisbane, Australia; b Centre for Policy Futures, Faculty of Humanities and Social Sciences, The University of Queensland, Brisbane, Australia; c Australasian College for Emergency Medicine, Melbourne, Australia; d School of Public Health and Preventive Medicine, Monash University, Melbourne, Australia; e Emergency Department, St Vincent's Hospital Melbourne, Melbourne, Australia; f Emergency Department, Colonial War Memorial Hospital, Suva, Fiji; g Global Programs, Emergency & Trauma Centre, Alfred Health, Australia; h Emergency & Trauma Centre, Alfred Health, Australia; i Port Moresby General Hospital, Papua New Guinea; j Emergency Department, Vaiola Hospital, Nuku'alofa, Tonga; k Public Health Division, Secretariat of the Pacific Community, Suva, Fiji; l Faculty of Medicine and Health, The University of Sydney, Australia; m The Sutherland Hospital, NSW, Australia; n NSW Ambulance, Sydney, Australia

**Keywords:** COVID-19, Pandemic, Emergency care, Human resources, Health workforce, Healthcare workers, Pacific region, Pacific, health system strengthening, Health system building blocks health

## Abstract

**Background:**

This study explores emergency care (EC) and other frontline healthcare worker (HCW) experiences responding to the COVID-19 pandemic in the Pacific region. The crisis has reinforced the crucial role well-trained, resourced, and supported EC providers play in supporting vital health systems and services in all global regions not only during ‘business as usual’ periods, but in times of tremendous stress and surge.

**Methods:**

Qualitative data were collected from EC providers and relevant stakeholders in three research phases in 2020 and 2021. Data on the World Health Organization's (WHO) Human Resources Building Block, adapted for the Pacific EC context, was thematically analysed. Key findings were further analysed to identify enablers and barriers to effective EC pandemic management.

**Findings:**

116 participants from across the Pacific region participated in this study. Five themes emerged: (1) EC providers performed multiple pandemic roles; (2) Importance of authorities’ valuing frontline HCWs; (3) HCW mental health and exhaustion; (4) HCW tension managing stigma, personal/professional expectations, and chronic health needs; and (5) Building health and human resource capacity.

**Interpretation:**

This study significantly contributes to the limited scientific literature on HCW experiences responding to COVID-19 across the Pacific. Recommendations arising out of this research align with consensus priorities and standards that were identified pre-pandemic by health stakeholders across the Pacific for enhancing EC system development. With limited HCWs available for many Pacific nations, it is imperative the dignity and welfare of local HCWs is genuinely prioritised.

**Funding:**

Epidemic Ethics/WHO, Foreign, Commonwealth and Development Office/Wellcome Grant 214711/Z/18/Z. Co-funding: Australasian College for Emergency Medicine Foundation, International Development Fund Grant.

**Research in context**

**Evidence before this study**

Human capital for health is the core of a well-functioning health system: people are key to overall building block interconnectedness, and community is an important part of the functionality of robust health systems and resilient pandemic response in Low- and Middle- Income Countries (LMICs). Following extensive literature reviews (SCOPUS, Google Scholar, WHO resources, Pacific and grey literature) we found application of the emergency care (EC) Systems building block framework to the Pacific context. This identified several consensus standards (10 out of 24) and priorities pertaining to Human Resources and Training. This reflects important current workforce gaps highlighted by limited formal post-graduate training for EC and lack of permanent staff in EC facilities, which hinders health worker performance through lack of training, knowledge and motivation, further complicated by care delivery in overcrowded and unsafe environments. Data on EC provider and other health care worker (HCW) experiences responding to COVID-19 in the Pacific context is scant.

**Added value of this study**

This prospective, qualitative study explores and captures the rich and diverse voices of EC clinicians in the Pacific region and documents lessons learned to inform recommendations to improve health system preparedness for future public health emergencies. We confirm that EC providers as experienced innovation and decision-makers are central to health system response to COVID-19.

Mental health concerns working throughout a pandemic were confirmed as being further exacerbated by workforce shortages, being stretched beyond capacity, limited access to mental health services and the experience of isolation, stigma and discrimination from colleagues, family and community. Our study illuminated the need for Governments to adopt holistic approaches to providing a safe and supportive work environment that meet the needs of both individual HCWs and those of their families and communities. More specifically, this paper has unpacked the barriers and enablers to ensure EC providers are valued and integrated into effective multi-sectoral response.

**Implications of all the available evidence**

Documenting and supporting vital human resources for health during the COVID-19 crisis is not only crucial for improving the safety and wellbeing of EC providers and HCWs as the pandemic continues to unfold, but for future public health emergency planning. Ensuring sustainable human resources for health is also critical for protecting fragile and underserved healthcare systems and services in LMICs. It is especially important that the pandemic not completely undermine the hard-fought gains many LMICs have made to progress global health equity and Universal Health Care coverage (UHC) in recent years. This Pacific regional expertise can be leveraged for valuable learning for national and international actors to support the development of robust and sustainable EC system via increased investments in training, education, and health workforce safety and well-being. This is of relevance to LMICs where health and human resources and training are an intersecting priority for sustainable development, emergency and disaster planning and management, and effective climate change response.

Alt-text: Unlabelled box

## Introduction

*COVID‐19 has exposed a great paradox of health care—who protects the people who protect us? While every death is a tragedy, health care workers (HCWs) are at significantly higher risk of infection than the general population, and illness and death among HCWs impede delivery of essential health services. The costs of solving this problem must be borne not by individuals, but by the societies that they serve*^1^

**Figure 1:**
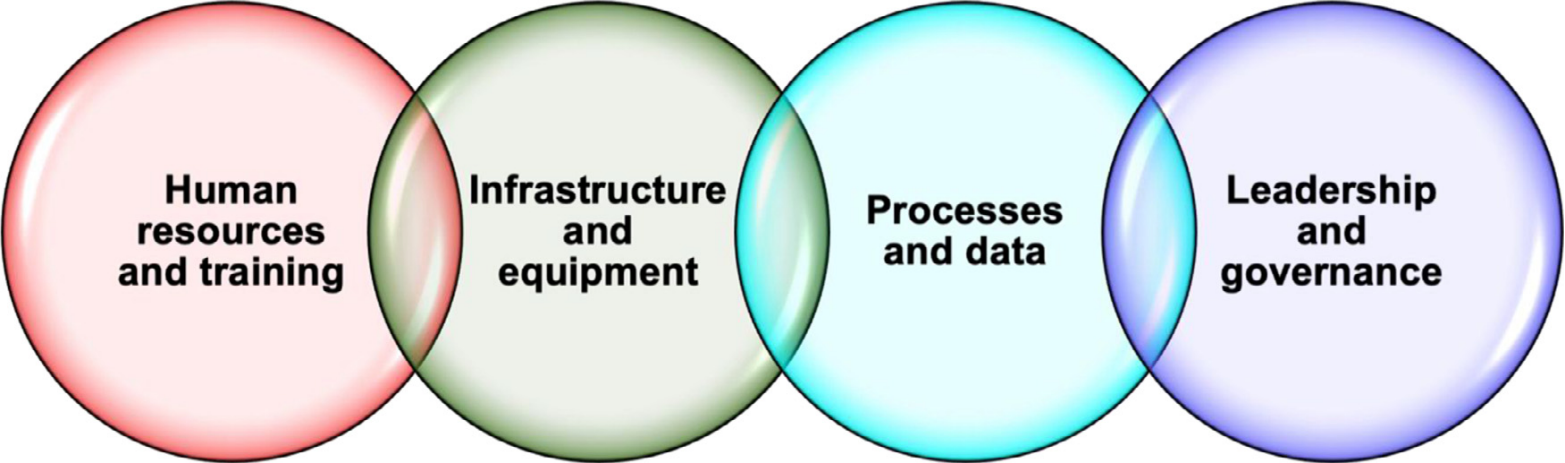
WHO health system building blocks, adapted for the Pacific EC context and this qualitative research project.

The World Health Organization (WHO) defines the health workforce as “all people engaged in actions whose primary intent is to enhance health”.^2^ In terms of health systems strengthening, WHO places human capital for health at the core of a well-functioning health system: people are key to overall building block interconnectedness, and community is an important part of the functionality of a robust health system. Importantly, EC stakeholders in Pacific Island Countries and Territories (PICTs) have also identified the development and support of human resources and training as the most urgent or priority building block for safe and effective EC systems in their region. Like the WHO, PICT EC stakeholders agree that although health workforce numbers and capacity is crucial, the human resource building block intersects with all other EC building block components within a complex adaptive system (Figure 1).^3^ This is of relevance to PICTs where health and human resources and training are an intersecting priority for sustainable development, emergency and disaster planning and management, and effective climate change response.

However, prior to the COVID-19 outbreak, most PICTs did not have formally trained or permanent staff in their EC facilities. Further, very few PICTs reported any trained staff for pre-hospital care, which forms the foundation for all EC.^4^ In a pre-pandemic study involving over 200 EC stakeholders from 17 PICTs (August 2018–April 2019), participants recommended standardised regional EC training, staff recruitment, specialist recognition and safe working hours for 24-h EC provision.^3^ In that study, training for resuscitation, triage and pre-hospital care were identified as three priority areas for EC providers; necessary training for effective pandemic, emergency and disaster response.

Certainly, the subsequent COVID-19 crisis has served to highlight the significant role well-trained and supported EC providers and frontline HCWs play worldwide in supporting vital health systems and services operating in times of tremendous stress and surge. ‘Healthcare personnel-centric’ COVID-19 care policies – aiming to reduce risk of HCW infection, ensure HCW welfare and safety, and improve HCW willingness to present for duty – are lauded as complementing traditional ‘patient-centric’ pandemic management approaches.^5^ Reports from Africa,^6^ the Middle East,^7^^,^^8^ Asia^9^, ^10^, ^11^ and Latin America and the Caribbean^12^, ^13^, ^14^ reinforce how HCWs operating in diverse systems and settings are putting their physical and mental health and wellbeing – and lives – on the line to counter COVID-19 outbreak, transmission, morbidity and mortality in local populations. The ethical, place-based tensions, stigma, and moral conflict overstretched HCWs face are increasingly documented.^8^^,^^15^^,^^16^ Such tensions can contribute to acute insomnia disorder to suicide among frontline health personnel.^17^^,^^18^ In addition, frontline HCWs are more susceptible to developing PTSD based on studies examining emergency department personnel and other HCWs responding to SARs and MERS disease outbreaks in 2003 and 2015 respectively.^19^^,^^20^

It follows that the data on EC providers and other HCW experiences responding to COVID-19 in PICTs, as well as in the broader Pacific context and neighbouring South Asia region, remains piecemeal and limited. For example, a 2020 study in Papua New Guinea examined the barriers and enablers impacting frontline HCW successfully swabbing and testing for COVID-19. That study found inadequate staffing and too few staff trained to swab, while HCW stigmatisation associated with their wearing of PPE was further documented.^21^ Another reflexive study examining the lessons learned from repatriation and compulsory hotel quarantine of 1522 Vanuatu citizens and residents to that COVID-19 free Pacific Island country in May–June 2020, highlighted insufficient health staff numbers and the need to strengthen coordination of health operations between Vanuatu's Ministry of Health and provincial health offices to avoid replication and undue managerial burden.^22^

Documenting and supporting vital human resources for health during the COVID-19 crisis is not only crucial for improving the safety and wellbeing of EC providers and HCWs as the pandemic continues to unfold, but for future public health emergency planning. Ensuring sustainable human resources for health is also critical for protecting fragile and underserved healthcare systems and services in low and middle-income countries (LMICs). It is especially important that the pandemic not completely undermine the hard-fought gains many LMICs have made to progress global health equity and Universal Health Care coverage (UHC) in recent years.^23^ This is equally important for countries and territories in the Pacific region.

Therefore, this study explored the voices and experiences of EC and other healthcare personnel at the frontline of the Pacific region's COVID-19 response through the lens of the WHO's second health systems building block, Health Workforce (human resources for health), adapted to the Pacific context.^3^

## Methods

### Study design

The study methods are described in detail in another paper in this series.^24^ In brief, this study was conducted as a collaboration between PICT and Australian researchers, and employed prospective, qualitative research methods grounded in a phenomenological methodological approach.^25^^,^^26^ Data were collected from EC providers and other relevant stakeholders across PICTs in three phases between March 2020 and July 2021 (Table 1). Informed consent was obtained from research participants through a mixture of written and verbal consent. Semi-structured interview and discussion guides developed by the research team were used in Phases 2 and 3.

**Table 1:** Three qualitative data collection phases March 2020–July 2021.

**Phase 1**	Online support forums via ZOOM	• 13 online support forums hosted by SPC and ACEM, between March and October 2020 • > 80 active participants (EC clinicians and stakeholders) from PICTs (and some non-Pacific countries) voluntarily engaged in online discussion
**Phase 2**	In-depth interviews via ZOOM	• Semi-structured interviews (45–90 min) with 13 key informants in Fiji, Kiribati, Palau, Papua New Guinea, Samoa, Solomon Islands, Timor Leste, Tonga and Vanuatu • Purposively selected: key informants coordinated EC in a PICT during the COVID-19 pandemic
**Phase 3**	Focus group discussions via ZOOM	• Three focus groups, with EC stakeholders from Pacific regions of Micronesia (Federated States of Micronesia, Kiribati, Marshall Islands, Nauru, Palau, the northern Pacific states), Polynesia (Cook Islands, Samoa, Tokelau, Tonga, Tuvalu, other Small Island states) and Melanesia (Fiji, Papua New Guinea, Solomon Islands, Timor Leste and Vanuatu)

Data collected at each phase were digitally recorded with participant permission, transcribed verbatim, and subsequently de-identified to protect participants’ anonymity. All data were preliminarily coded using QSR NVivo15 using a hybrid inductive (data driven)^27^ and deductive^28^ approach. Deductive codes were derived from the WHO health system building blocks adapted for the Pacific EC context (Figure 1).^3^ The subset of data related to Human Resources and Training was then thematically analysed by authors CEB and SK. Emerging themes and tentative findings were presented to the broader research team at several online meetings for verification through discussion and data triangulation. Thematic findings were further analysed to identify enablers of, and barriers to, effective EC responses to the COVID-19 pandemic related to human resources for health in the Pacific region.

Ethics approval was provided by The University of Sydney Human Research Ethics Committee (Reference 2020/480) and registered with Monash University Human Research Ethics Committee (Reference 28325). Research protocols for Phases 1 and 2A of the research were also reviewed by the World Health Organization's AdHoc COVID-19 Research Ethics Review Committee (Protocol ID CERC.0077) and declared exempt. Reporting of study data adheres to Enhancing the Quality and Transparency of Health Research (EQUATOR)18 and Standards for Reporting Qualitative Research (SRQR)19 guidelines.

## Results

In total, 116 participants from at least 14 PICTs, participated in this study. Five themes emerged relating to human resources and training:

(1) EC providers performed multiple roles during the pandemic.
(2) Importance of recognising and valuing frontline HCWs by authorities.
(3) HCW mental health anguish and exhaustion.
(4) HCW tension managing stigma, family and cultural expectations, and chronic health needs.
(5) Building health and human resources capacity.

These themes are presented in detail below. Table 2 also presents a summary of the synthesised enablers and barriers to effective pandemic response found within each of these themes, with illustrative quotes.

**Table 2:** Enablers and barriers to human resources and training in the pacific region for improved COVID-19 response.

Enablers	Barriers
Theme 1: EC providers performed multiple roles during the pandemic	
**EC provider inclusion in local response** *“The emergency staff are leading transport. They're on all the care committees.* [] *The national committee. And they're actually the people putting forward good evidence-based care and trying to override political decisions, as opposed to health science decisions. That's good”*	**Overriding clinical expertise and opinions** *“The current situation is, what the Director-General says is what goes. So we've been working really hard to get him to see the clinicians’ point of view. But usually if he fancies something he'll be like ‘No, we'll do this’”*
**EC acknowledged as a respected discipline** *“It's 100% emergency department now and it is recognised by the other departments this time, [] people are recognising what the teams are doing.* [] *I know there is still plenty of work to be done. But just being able to sit at the taskforce as a rep from emergency and the only other rep is from internal medicine because of infectious diseases.* [] *The team we are having now, I can see a brighter future”*	**Disregard and lack of understanding about EC** *“Emergency Medicine is not accepted in the country where they think ‘What is that?’, when I started”* “They don't really know how important emergency medicine is yet. Not unless, you know, they come in with an out-of-hospital cardiac arrest and wake up, for a bit”
**EC providers have extensive experience with ethical decision making and the allocation of limited resources** *“Ethical considerations – I think most importantly we needed to be informed about COVID, how aggressive it was, who it was infecting and what we could realistically treat. The issues then come down to really being realistic about what we can offer and what we can't.* [] *In countries where we have limited resource we need to be quite frank with patients and not set unrealistic expectations.”*	**Limited or lack of pre-existing training for disaster response** *“We don't in our training have much exposure to disaster management and so* [] *it was quite challenging to lead the COVID response and to get the systems up and running.* [] *I had to micro-manage everything: setting up the triage, the pre-triage… Having to micro-manage everything was probably the most difficult thing for me in the COVID response”*
**EC providers as experienced innovators and decision-makers** *“The other positive thing was that they* [EC personnel] *were very strategic in getting resources... Before* [we] *actually went into lockdown, they were ready with their proposals of what's to be done, where they needed help, how much money* [was needed] *to build, to refurbish the staffing quarters and COVID ward. They had a proposal ready for the* [visiting] *Prime Minister to endorse. They actually got everything they wanted. they were able to liaise with the highest authorities* [and] *get military people to come in to actually build and refurbish the facility. They were able to get the Ministry of Infrastructure to come and do the mending of the hospital wards”*	
**Team based nature of EC** *With emergency medicine it's not so much what the doctors do and the nurses do, it's more of a team, what we do together. that system is working for us in our department. It's being noticed by the other specialties and they're trying to see how they can make* [their team and processes] *better. There's positive impacts”*	
Theme 2: The need for authorities to genuinely listen to and value HCWs at the frontline	
**HCW acknowledgment and support at a national level** *“And I asked them* [nurses] *to have a meeting and they came up with a list of things that they would like addressed. And a lot of them were around their own safety, safety for their families, they wanted to know if there was, if the government would provide some sort of insurance should they die in the process of looking after COVID patients... after the nurses had their meeting, they brought the list of petitions for us to address. And most of them were around their own personal welfare. I sit on the national meetings, I was able to bring up their concerns nationally”*	**Lack of communication, engagement and consultation with HCWs** *“The staff, I think, are potentially one of our biggest problems They're really unhappy. They're frightened. They haven't been engaged with. When I talk about our staff, I don't mean the ED, I'm talking about hospital-wide. There's a lot of fear. I think it's not helped by, the government here keeps promising extra payments to them, there's no transparency around that, they're not going to get their extra payments. I think that's going to cause a lot of problems. Staff are already saying that they're not going to come to work when we get positive cases. That's one of my big fears”*
**Timely compensation and acknowledgment of hazard work** *“I can answer from the nursing side, which seems to be suffering a lot more than the medical side. Aside from the allowances a lot of the nurses aren't being paid their expected rates anyway, because of the circuitous public circus. So, I think that if they could at least get those raises that they were expecting, apart from the allowances, that would be a big help. But that's a huge process”*	**Inequity in risk allowance rates across sectors and nationalities of staff** *“I think the biggest problems we have faces is when they declare a state of emergency, the staff – should be paid – allowances, extra pay* [] *but none of the staff have received any allowance. Other government departments, l police, they're getting their allowances. And senior people within the* [hospital] *management are receiving allowances. Which everyone's fully aware of”* *“*[] *I didn't claim any extra allowances for extra duties because, on a personal note, we were really highly paid compared to the locals. And for to me to actually claim any compensation was unethical in a sense”*
**Respect and trust for clinical authority** *“Other strengths that stood out was how they used infection control, mainly, as their main advisor to the stakeholders’ committee. They did, because the thing is that, they knew they were not experts, and because it was run by nurses – that was the good thing; they didn't undermine nursing input, it was supported”*	**Executive direction without consultation** *“The leadership in the health department is poor. They lack guidance by clinical, subject matter experts, and* [when] *they get one, like, an international fix solution,* [they] *try to just implement it without contextualising it ... It's hard to trust most of our leaders”* *“I wish the government can trust its professionals to provide them with advice to make decisions based on their knowledge and they have the capacity. It seems like it's always a political move…”*
	**Absence of political support for clinicians** *“If there's anything that I've learnt from this* [it's that] *you really need the right* [political] *people throwing their weight behind something like the COVID response to get things done. Otherwise, you're just going to be fighting an uphill battle and it's frustrating”*
Theme 3: HCW mental health anguish and exhaustion	
**Regular HCW peer communication** *“The nurses are having another meeting now. It's the second since this week. There were a lot of things done, but the day after the first case was announced, there was a lot of panic and chaos. Now people are trying to be more focused. There have been quite a lot of meetings going on”*	**Impacts of compulsory clinical work** *“They're stressed & anxious, but so far none of them have come up with any major symptoms… we're all required to turn up for work unless we're sick. Everybody's at work.*[yes increase in sick leave]*…I actually had to call in, well not sick, but I actually told them I was sick from last week, I took 2 days off to recover and came back again”* *“I did find it challenging, especially initially with COVID;* [] *the pressure to ensure that things were up and running. We were pressured to work long hours to ensure that things were delivered and whatever was required was provided to the emergency operation centre”*
**Timely top-down information sharing** *“Initially, when the pandemic was announced, there was a lot of fear amongst the staff…. We tried to ensure that we had our weekly sessions of IPC, handwashing & how to do and how don't to do stuff with the different levels of PPE, to ensure that all the staff underwent that training. And we also had a presentation, we requested a presentation, from the MoH advisor regarding COVID. That was done quite early on….”*	**Social media misinformation exacerbates fears** *“But when we actually looked at many of the concerns they* [the nurses] *raised* [in a petition]*, they were around a sense of quote-unquote ignorance, over the disease, what was happening, and all the media, social media posts, showing how many doctors and nurses are dying elsewhere. That has been our biggest challenge, just to get the nurses onside”*
**Access to education, training and research** “*Everyone they were very scared because they had one positive case, everybody fled from the hospital and health centres. They didn't know how to wear the PPE, they didn't know about hand hygiene and respiratory hygiene. I was called to go out and assist in training, in different sites* [] *while there, we trained healthcare workers on PPE, how to wear them, how to do donning and doffing, also on hand hygiene and respiratory hygiene. After the training, it was very exciting to see the healthcare workers smiling,* [] *they were happy and confident”*	**No human resources for surge capacity** *“I will step up if I am required on issues around quarantine, where no one wants to go. They call me in the middle of the night, and I'm up and get there and sort things out for them, which is what emergency physicians do all the time”*
***Motivational and transparent leadership*** *“I'd say good leadership, from both the admin level, administrative staff, and also in terms of the emergency department. Our HOU* [Head of Unit]*is very open to communication and dialogue with everybody. There's no hiding of information. If it's available it's widely disseminated to everybody, there's no hidden agenda so to speak. People supporting people, healthcare workers looking out for each other…”*	**Absent network of support** *“It's just like your immediate supervisors who were kind of not listening to you. And then you have to think of some way out again.* [Tearful] *It's really, really difficult. It was just like, nobody was there for you, that's all. You just have to think, think, think and do things on your own. And the smartest way you can to bring things in for the good of your staff”*
**Workplace safety culture** *“When the first suspected case came through, I remember there was a lot of apprehension as to who's going to do it. I said to two of the senior nurses ‘We have to do the first case, we have to set the example – we need to go in and swab that patient because if we are scared to do it then everyone else is going to be scared in our team. We need to show them that it's okay’. I had to make sure that I understood, that I was clear in my mind, about how to don and doff, and what was the risk … Speaking to the network of emergency physicians helped me quite a lot to allay my fears and to ensure that things were done in a systematic, orderly way”*	
**Professional duty of care** *“I think that's also cultural here, in the* [country] *culture, and also in the* [] *faith, there is good works and doing your duty and honouring your fellow workers, teacher and country, is very high. People may be fearful, but that come to work, to play their part, certainly in the emergency department”* *“There were a lot of things I was unsure of. If I'm going to die what insurance was I going to get? There was nothing clearly defined on what my work is, what's the weight, what hazard allowance should I be getting for this?* [If I test positive for COVID-19 and] *I get separated, I'm not going to see my family for the next two potentially three to four weeks or much longer. Those were the things I was left in the dark and not sure* [about] *… However, I also knew we had a duty of care to our patients. All doctors are called they have a duty of care, so I said to myself I have a duty of care”*	
Theme 4: HCW tension managing stigma, discrimination, family and cultural expectations, and chronic health needs	
**Provision of alternative accommodation** *“People may be fearful, but that come to work, to play their part, certainly in the emergency department. We do find though that they're fearful of going home. And they're fearful of infecting family. And to that end, 90% of our staff now live in hostels near the hospital or in apartments donated by donors. There are only a few of us who still go home at night. That's one of the ways of cutting down anxiety is knowing that your family is safe from you”*	**Fear of infecting family** *“Staff were basically concerned about their children and their family at home, that was their main concern”*
**Provision of support for all tiers of staff** *“…And staff meaning from the doctors right down to the cleaners, they all come under you. And they have families whom you have to consider, so their safety is of paramount importance. Even the clerks too. So yeah, that's how I see it, you kind of take care of everybody who's working in your department”*	**Gender specific challenges** *A Pregnant HCW “had to separate from family” [quarantine] “& she almost got into a stage [of] self-harm. It's very challenging”* *“I was told that some of the female staff, they were willing but then they had received advice from their relatives at home not to partake [] in terms of participation, sometimes because of our culture, most cultures with male-dominated society they tend to listen to what the male relatives tell them to do”*
**Access to mental health services introduced and actively encouraged** *“We address this before the actual second wave, but I think at this point in time we may need that mental health team to come in to, [], do morale boosting sessions again, just to make sure everyone is – especially, everyone involved, from nursing to medical to the support staff as well”*	**Awareness of COVID-19 outcomes** **“***It's because they are exposed to all the negative and severe cases and the fear is real for them.* [] *You would rather not know and be ignorant of the severe cases… And then at nights when we have a resus or when we have a patient on oxygen and everything is quite noisy with the monitoring and beeping and all of that, you are a* [HCW] *and you're lying two cubicles away and you can hear the beeping then you hear the distress alarm go off and you know exactly what's happening and then it plays a role on you mentally as well”*
**Staff commitment and motivation** *“Staff commitment, staff attitude, and strong leaders below me. As the Incident Manager I had leaders in surveillance and clinical care and all of them went above and beyond their responsibilities to get SOPs [*Standard Operating Procedures*] and other functional documents, and teach those things like treatment guidelines to the staff”*	**Social and cultural pressures** *“A lot of the nurses, when they come in and they do the training, they understand it* [COVID-19 risk]. *But then they develop pressure from their families, their families say, no you shouldn't be working or no we have a positive case you shouldn't go in. It's an important opportunity to have* [family forums]*. The nurses want their families to hear it from us. Because when they tell their families, they don't seem to pay much attention”*
Theme 5: Building health and human resource capacity	
**Commitment to the discipline of EC** *“I just looked at it as a* [opportunity], *I just kept asking a question to myself when I came to the emergency department. I saw this department as the worst department within the hospital. I think it is the same everywhere else. But whatever small contribution I can give that will be my driving factor, to get this department into a better state than when I started off.* [] *Whatever small contribution I can do with a small young team, I think we can build on this in getting the department. I think COVID just came in as a bonus I would say to learning how to better prepare us for the long run. That's how I would see it”*	**Limited prior investment in EC systems** *“I think the downside of it was that I don't think as a country we have faced a threat this big, and the fact that the* [local Pacific Island country] *Health System is very immature. They don't have capacity at all levels, so they were not really prepared for a major threat like this”*
**Rapid capacity development of EC personnel** *“The emergency staff have really taken the forefront of planning, to the extent that they've actually argued with national health ministers, which is great”*	**International HCWs returning home** *“To be honest, the nursing team are really down, because physically they're down with manpower as well because we lost* [international] *nurses, who were emergency nurses, specialist nurses. And then we requested to management if they can replace within the national emergency nurses, but it hasn't come forward”* *“Most of the staff here are imported”*
**Multi-sectoral and multidisciplinary response/training** *“Now more and more people are coming to be part of the team, both medical and non-medical. You realise that some of the tactics that you would use in the ED to teach may not be so good for people that are not medical. You learn to change your approach.* [] *Now we get to work with all these other people, these CEOs, who are also part of this team”* “*One of the other things we realised is, not just the medical staff, the non-medical staff also play an important role, especially in EC, like our security guards, our ward assistants and orderlies… they're also very important... It's important they are protected because they are also part of our team.* [pandemic] *training* [should not] *be isolated to one group but* [] *accessible to everybody*”	**Lack of available, willing, or trained HCW back up** *“In return they gave us novices.* [] *We have to start all over and train, not at this point in time, especially in this disaster. I call it a disaster”* *“I think medical staff see it as their duty, as their honour, and get in and do it. However, I should say that the non-ED people – so we've seen this in the surgeons and the orthopaedics – if their elective surgery has cut down, they're very happy to stay at home, rather than go and help in other areas”* *“…even though we received those ventilators machine, we needed to have training. I don't think most of those people here* [] *especially the doctors including those working in referral would be able to use that ventilator”*
	**HCW and non-clinical staff contracting COVID-19** *“We had 9 nurses that went down with viral symptoms, and they required screening. Our challenge here is that our lab staff were also infected. Testing capacity is the problem”*

### Theme 1: EC providers performed multiple roles during the pandemic

**Figure 2:**
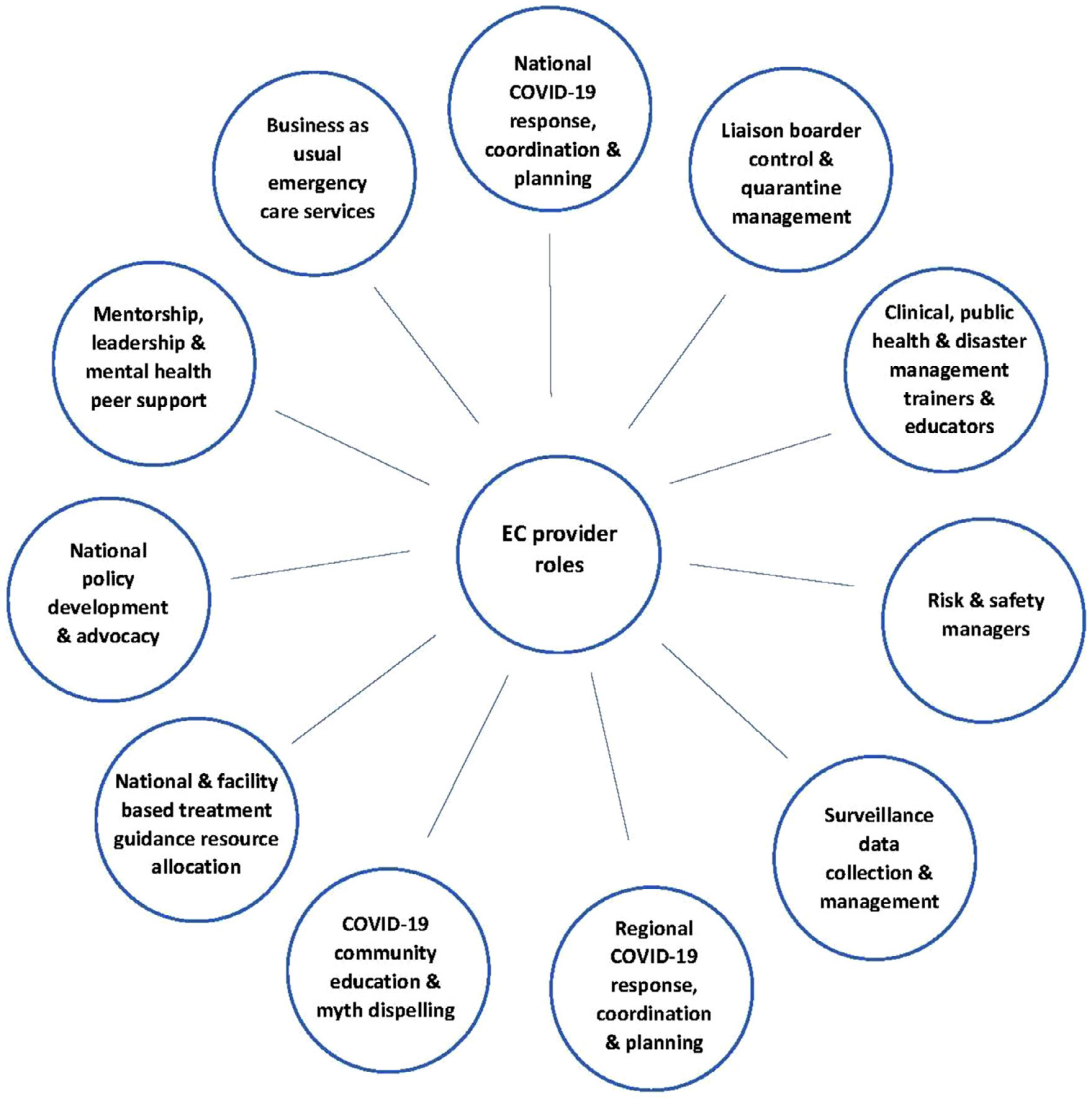
The multiple roles held by EC providers in a pandemic.

Study participants explained that, even in advance of the pandemic, they undertook multiple roles and were already at capacity. However, the COVID-19 response was stretching them to another level. Participants became motivators, advocates, mental health peer-supporters, safety advisers, trainers, educators, and emergency leaders and strategic planners in the health sector and beyond (Figure 2).

Participants explained that at the pandemic onset, EC providers were willing to assume local leadership roles. But the *“panic”, “fear”*, and *“chaos”* should not be downplayed. As an EC doctor put, *“It would be true to say everyone is shit scared”*. Participants recognised that iterative training and teamwork were crucial to allaying EC and broader HCW fear. Cultural resilience mixed with religious faith played a role.

*“In spite of the fear we are doing okay* [] *A lot of our culture comes through in our work. Working together, the cultural concepts* [] *like love of country, love of people”*

Nurses spoke of assuming senior roles for many reasons: their clinical leadership and understanding of health systems and communities; lack of doctors; need to backfill unwell staff and foreign HCWs who had left.

*“I didn't have my senior medical officers on the ground, so I had to play that role of senior person to liaise between the staff and management* [for] *all kinds of things happening in the ED. Patient deaths, moving patients, transporting things* []*, pushing for enough PPE* [] *I stepped in both for the medical and nursing teams”*

Even if medical registrars (doctors in speciality training) were present, nurses discussed how registrars looked to their experience.

*“I had very junior registrars who didn't feel their voice would be heard. They kept on coming through me to do all the talking for them. The management was listening to me”*

Consequently, nurses called for a *“conscious effort”* to include their knowledge leadership in high-level pandemic decision-making, alongside doctors.

The efforts of EC providers motivated HCWs working outside of their departments: *“We emphasised* [] *if you don't stand up to help then who will? If everyone doesn't want to be involved, then who's going to do it?”* Participants agreed that health and other sectors looked to them for leadership, reassurance, education and technical support:

“[Pre-COVID] *it would mostly be physicians and nurses* [who ask for our help]*.* [Now] *a lot of our border patrol folks, people at immigration, customs, biosecurity”*

Behavioural modelling became an important part of HCW's role: *“If we portray fear, they too will be scared”*.

The handful of senior EC providers were further required to establish infrastructure and supply chains in tremendously challenging settings.

*“We set up the field hospital in* [regional area] *to create space. We did that with no funding initially*”

Along with the recruitment of lay volunteers who were rapidly trained on *“basic things”*.

EC leaders demonstrated innovation by using radio and digital platforms to refute misinformation and calm community, upskill staff and improve supply chain, triage, and patient flow processes in remote locations*: “I created* [an online group] *because at the end of the day the burden comes to us; there's no other choice we have to develop something to help”*. To reassure HCWs’ family members and improve morale, EC doctors held family forums: *“Nurses want their families to hear it from us”.* In other contexts, educating the traditional leaders was a priority: “[To] *ensure they're on the same page as we are* [for] *when they talk to the community”.*

Throughout the unfolding pandemic, EC personnel became fearless champions “f*or the good of* [our] *staff” and those* with cultural status leveraged their *“invisible hierarchy of importance”* to advocate “*for the good things in ED”*; for example:

*“I care a lot about my staff* [] s*o advocacy is a natural role for me. I don't pull any punches, I told them straight* [the major leaders]*. At one stage one was like ‘Oh here comes Dr Know, he knows it all’... I just laughed it off. I said, ‘Yeah, well, expect another ear bashing’.”*

Participants further spoke of HCWs becoming risk managers and safety control officers, again utilising digital and online communications channels.

*“We send out daily safety messages to the* [online] *group, to remind people to be conscious of safety,* [] *of what the risk issues are”*

In the absence of or in complement to formal mental health services, many EC leaders became mental health and wellbeing peer-supporters and advocates: *“I support them online, and call them, and support mentally”*. The need to provide mental health supports to colleagues in countries where COVID-19 had not yet emerged was raised.

*“Even though we don't have COVID cases, the cost of keeping our islands free of COVID is taking a toll on all* [HCWs]*… We've had people under* [COVID-19] *investigation and the whole island goes crazy… So, we regularly schedule* [mental health sessions] *for our workers, to ensure everybody remains sane … Sometimes our leaders question ‘Why do you spend this amount of money?’ We told them … ‘If you take care of your people you get the work done; you don't then nothing gets done’… We don't have the luxury of a lot of* [HCWs]*”*

### Theme 2: Importance of recognising and valuing frontline HCWs by authorities

Study participants identified the urgent need for government to overtly value HCWs – courageously stepping up in multiple roles to lead and support countries’ pandemic response and overall security (Theme 1). Participants identified diverse ways this should occur but suggested the main way being timely incentive and allowance: *“Staff were concerned that if we're doing a risky job,* [they] *should get paid certain allowances for it”*

Compensation was pressing. Participants explained that many HCWs were breadwinners in large households and if they die their families need support. Additionally, many had lost financial supplements from their private hospital or consultancy work that abruptly ceased due to clinic closures and/or government demands taking precedence. Removal of pre-pandemic overtime payment to nurses in some countries was considered particularly problematic.

Participants gave examples where others were paid COVID-19 allowances but not HCWs. Government inconsistency was causing *“huge problems”* for HCWs in pandemic management.

*“Other government departments, like police, they're getting their allowances and some senior* [government] *people. Which everyone's fully aware of. That's caused a huge problem. It's immeasurable. Staff are really angry, they're not coming to work… It's something we can't do anything about”*

Pay discrepancies among local HCWs and those paid higher salaries by international agencies and donors became another source of demoralisation and tension: *“The difference in remuneration is discouraging... The locals are resentful”.* Due to lack of parity in allowances, poor wages or overtime cuts, many HCWs in this study rely on civil society and community donations.

*“Having donors provide food every day* [and] *petrol for your car is not a blessing, it's a necessity to keep doctors and nurses here working. They're not able to earn extra that normally would make up the bulk of their monthly pay”*

EC leaders further discussed managing HCW commitment when staff were not being paid in a timely manner: *“My staff, they don't get paid for 3 months …* [] *It's terrible”.* Some participants were particularly frustrated when authorities promised additional staff payments that were not forthcoming: “[Staff know] *they're not going to get their extra payments and that's going to cause a lot of problems”*. Participants highlighted similar issues among staff when authorities promised PPE and other medical supplies that did not arrive. Participants emphasised that in low-resource settings “*the small things do matter*” for staff welfare and dignity including *“cake and food”* and *“laundry”*.

Government unwillingness or inability to listen to HCW requests and/or provide timely compensation or non-monetary reward was distressing for many.

*“My main concern was the compensation* [for our HCW staff] … *None of my authorities can answer me, they just push me away from asking. I hope they're on this session so they can hear my voice.* [] *As a nurse that works 24 hours, it's very risky* []*. We're not asking too much.”*

Disparity in reward between nurses and doctors caused tension, i.e., nurses receiving comparably poor quarantine accommodation, poorer meals, and lack of access to counselling: *“It was disturbing to our staff and people were very frustrated”*.

### Theme 3: HCW mental health anguish and exhaustion

The pandemic's impact on EC and HCW mental health across the region was another prominent theme. Multiplicity of HCW roles, lack of valuing by government, chronic HCW shortages and long hours, lack of PPE and equipment, combined with the fear and anxiety for themselves, their families and communities (Themes 1 and 2), made for HCWs feeling *“exhausted”, “burnt out”, “angry”, “sad”, “scared”, “overwhelmed”*, and *“mentally really, really affected”.* Exhaustion and poor mental health understandably resulted in staff taking sick leave. However, this was challenging to manage with pre-existing staff shortages in isolated and underserved environments.

*“Yesterday, we had eight of our nurses that went down with the flu. I'm not sure if it's really flu or the mind playing up. I can't blame them.”*

Quarantine away from family and community also exacerbated poor mental health, especially for participants living in communal cultural settings: *“Those returning from quarantine were not good* [and] *some developed depression, anxiety”; “*[In my country] *everybody seems to smile and be happy about everything but when I got back* [from quarantine] *I couldn't”*. A participant elucidated how a pregnant HCW *“had to separate from family and she almost got into* [self-harm]*”*. Another observed that hospitalised HCWs with COVID-19 struggled more compared to non-health workers: “*because they're exposed to all the negative and severe cases and the fear is real for them”*.

Although HCWs described themselves as resilient having faced many natural disasters and infectious disease epidemics (including coinciding dengue and other disease outbreaks), there was widespread acknowledgement that the pandemic's mental health impact was unprecedented.

*“Burnout is almost daily.* [HCWs] *in tears. That's when I knew we are treading a fine line and managing people on that fine line.”*

Participants agreed addressing HCW mental health needs – *“Whether you like it or not we have to look after ourselves”* - had never been at the scale and urgency they were witnessing everywhere.

Another participant explained that in their Pacific Island country, storytelling and talking to build HCW confidence, trust and wellbeing was an enormous cultural strength.

Participants further described how they and others had spoken up, sought out and implemented supports from mental health specialists across the region (i.e., local mental health teams or from regional or international mental teams through online channels). When discussing the benefits of accessing mental health services, one summarised:

*“It gives them* [HCWs] *a positive way of looking at things.* [] *It gives a sense someone cares, someone listens to us… It helps a lot”*

The above participant then described how patient care benefited when HCWs accessed consistent mental health supports in conjunction with repeated COVID-19 training.

*“When staff are comfortable about whatever walks through the door, whether it's COVID or other cases, there's less panic, less hysteria, and that translated into more trust amongst the patients* [] *coming to receive care.”*

While participants welcomed online mental health supports, issue surrounded accessibility.

*“Like everything else, everything is done online… We all know there's only a few people who are able to do it. The rest just cannot get onto good internet.”*

Finally, in the second and third phases of data collection, participants raised that COVID-19 vaccine introduction had helped many HCWs mentally: *“Now that we have the vaccine and we are all vaccinated, everyone is coming in, wanting to give a hand”*. However, others reported vaccine safety played into fear, anxiety, and poor mental health in some settings, including HCW reticence to be vaccinated.

Table 2 further summarises how information sharing, peer support, motivational and transparent leadership, workplace culture and relationships can act as enablers or barriers to mitigating the impacts of HCW mental health anguish and exhaustion.

### Theme 4: Tensions managing stigma, family expectations, and chronic health needs

Stigma was commonly experienced by HCWs at the COVID-19 forefront in the Pacific region. Following Theme 3, participants agreed stigma contributed to HCW poor mental health. They experienced or observed HCWs and non-medical staff excluding and avoiding health personnel working in COVID-19 screening, triage or with COVID-19 patients or suspected patients.

*“My nurses weren't allowed by the nurses from the other hospital sections to get in with them on the same vehicle for drop off”*

*“If we've walked into start our duty, some hospital staff will be whispering to others or even to the public and patients coming in, about who we are, what we're doing”*

Participants were shunned by family and pressured by households and community not to work. They noted this frequently occurred in countries with communal living and households with extended, multi-generational families.

*“I did have that labelling, ‘Oh you're coming home with COVID go outside’ ‘Have your own separate toilet’. I got that from my family. Some of my friends also decided not to see me”*

Not only HCWs, but non-medical staff faced exclusion.

*“The* [] *cleaner who was part of the COVID team when she went back to family… They refused to allow her into the house. Not only her family but the neighbours. She came back to quarantine crying…”*

In certain contexts, gender and/or cultural challenges exacerbated HCW stigma.

*“Some of the female staff, they were willing* [to work] *but they'd received advice from relatives at home not to partake… because of our culture, most cultures with a male- dominated society tend to listen to what the male relatives tell them to do”*

Some participants raised that in certain settings there was a real risk of homelessness for their younger female nursing staff with less household authority.

*“For the lower category of nurses living with other relatives, or married couples living with someone else, they don't have that same power within their house. Their* [households] *were like, ‘I don't want you here, you move out, go’”*

In turn, especially at the pandemic outset, HCWs similarly expressed feeling torn and distressed about working for fear of infecting family members: *“Staff were basically concerned about their children and family at home, that was their main concern”*. In one setting, staff shifted into “*hostels near the hospital or into* [donated] *apartments … That's one of the ways of cutting down anxiety is knowing that your family is safe from you*”. In other settings, authorities *“rent*[ed] *rooms at hotels so our nurses and doctors can go and sleep cause their family don't want them to go home… Some of the workers… were just so scared that they didn't want to take that chance”.* Several participants noted HCW colleagues often lived with and cared for elderly parents, many with comorbidities, and fear of transmission to their loved ones was *“a huge burden”.* Participants furthered that HCWs who experienced chronic disease were also anxious about working. HCWs who had maintained confidentiality around their health conditions *“suddenly”* disclosed these to management and staff.

*“All of a sudden we knew what people's conditions were. Before* [COVID-19]*, people don't go around telling people that they have hypertension or that they're diabetic”*

There was also discussion around the prevalence of chronic disease among nursing staff and having to manage pre-existing HCW shortages with this in mind.

*“It's very tricky. We found out that 50% of our nurses had background comorbidities. We're already short in numbers”*

However, in the second phase of data collection, some participants reported HCW stigma had lessened, citing HCW and community education and empathy.

*“Actually,* [] *there's more support, everybody seems to be texting each other, or on social media platforms and saying we're praying for you, thinking of you... There's a lot of moral support amongst colleagues as well. We all know that we're at risk of burning out, we're getting tired, and it's still in the middle of our surge”*

### Theme 5: building local capacity

Addressing longstanding HCW shortages in the Pacific region, including EC personnel shortages, was a priority for study participants. All spoke of stressful staffing issues, which pandemic preparation and response accentuated.

*“We're always short in staff. Some have migrated, retired, some died. It's something that we've been battling with throughout…This exhausts the remaining staff mentally, emotionally and physically”*

*“Our doctors and nurses are ageing, they're facing retirement and we can't get any new doctors in.* [PICT] *is a small country, so there's not big numbers in our medical schools… we cannot supply or replenish quickly”*

HCW demand had considerably intensified in all countries given local HCWs had to *“start vaccine rollout”* and *“run the hospital and public health for 24 hours and at the same time provide all this quarantine and isolation activities”*. This included *“heavy stringent document screening… in terms of exposure, potential exposure* [at different ports]*”* of seafarers on off-shore cargo and other ships, as well as performing *“double work”* with repatriation flights of sick seafarers and others to their country of origin. Participants raised even if rapid response systems were in place, or there were enough ventilators or PPE, such positives are undermined by a lack of staff *“either in numbers or in training”.* Populations in provincial areas and outer islands were identified as particularly vulnerable.

During the second phase of data collection, robust discussion emerged over Pacific health systems in some PICTs being *“really under-prepared”* for COVID-19 due to limited investment in localisation of EC capacity. Discussion focused on some countries having *“sort of trained”* local populations resulting in a reliance on politically nuanced external donor support that often focused on equipment over training.

“*I think a big lesson here is not to get sucked up into what people are offering, what donors are offering* []. *You need to know your health systems very well* [] *and be confident about, what I can do and what is appropriate, now, or in the future, and work along that*”

“*We need brave people who are able to say that, at this point of time, that is a waste of* [donor] *resources – yes, maybe down the line then we can go and get those – but let's put these basic systems into place and then we'll deal with that down the line”*

One doctor spoke of donor countries that were – in their view – *“still pulling strings in the background”* of limited benefit to the health and wellbeing of local populations, or the genuine support of local HCWs for strengthened Pacific EC systems.

Over-reliance on international personnel – contrary to increased investment in local HCWs - was symptomatic of the lack of localised investment in human resources and training that the pandemic inflamed. Most international HCWs reportedly returned or were recalled home before country borders shut: *“We lost nine Filipino nurses who were EC specialists”*. However, the arrival of newly graduated Pacific medical doctors from Cuba boosted local HCW numbers and morale. Participants spoke of the *“fortunate”* nature of their return. Others highlighted how neighbouring countries had managed to provide them with retired nurses who had undergone refresher training.

## Discussion

This qualitative study is the first to deeply capture the capacity and capability of human resources at the frontline of the EC and broader COVID-19 response in the Pacific region. Its five thematic findings elucidate the rich voices and diverse lived-experiences of local EC stakeholders to inform improvements in health system and health workplace preparedness for the unfolding COVID-19 pandemic, as well as future public health emergencies and natural disaster events so prevalent in this global region.^29^^,^^30^ Our study illuminated the lack of pre-pandemic investment in ‘business as usual’ EC systems with human resources and training.^3^ Key lessons learnt from this study are presented in Box 1.

Box 1 **Key lessons regarding human resources and training**

Ongoing investment in training and education of HRH as part of EC system strengthening is needed to progress localisation of public health emergency response

Investment in EC systems is essential for the attainment of Universal Health Care

The demonstrated competence of EC providers as leaders and collaborators highlights the importance of consulting them as part of local- and national-level multisectoral action. EC providers should be engaged to advise on the determinants of health through the application of feasible and cost-effective interventions that align with the ceiling of care offered in-country

Workplace culture and safety is essential to maintaining a healthy workforce. The needs for access to mental health services are vital in mitigating burnout especially in face of significant HRH shortages in many Indo-Pacific contexts.

Alt-text: Unlabelled box

For participants and HCWs at the frontline, the evolving COVID-19 crisis amplified the well-known need for long-term strategy and meaningful investment in the training and building of human resources for health *on the terms* of Pacific HCWs (Theme 5).^3^^,^^31^ Iterative strengthening of local EC skills (clinical and non-clinical) to work within interfacing health services and systems (multi-level, multi-sectoral and inter-generational) is especially needed. The COVID-19 experience has reinforced the urgent need to train, support, retain, reward and integrate EC staff in all Pacific health systems (Box 2). For this to occur, participants collectively called for country and regional authorities to listen to and include EC personnel (specialists, registrars and nurses) in high-level pandemic planning and decision-making (Themes 1 and 2), and to genuinely compensate exhausted, fearful yet committed HCWs for the *multiple roles* they are playing (and will continue to play) for the economic, social, and cultural survival of Pacific countries and communities *everywhere* (Themes 1 and 2). Indeed, our study found EC providers played vital roles as innovators, motivators and educators of HCW colleagues, traditional leaders and community dispelling myths around COVID-19 and vaccination.

Box 2 **Human resources and training recommendations for action**

Ensure access to sufficient and timely technical training of all frontline care providers responding to the pandemic including key HCWs and adjacent support staff

Ensure safe working conditions in emergency departments including psychological health and safety of all health workers

Governments implement programmes focused on the effective retention of the health workforce

Bundled interventions for HRH that provide, integrated training and career pathway opportunities, financial and non-financial incentives for ‘risky’ work

Integration of emergency care workforce requirements in disaster preparedness and response planning

Alt-text: Unlabelled box

Consistent with the handful of qualitative studies on HCWs responding to the COVID-19 crisis in LMICs (e.g. Colombia, Nepal, Iran, China, Indonesia, Brazil, Sri Lanka) and high-income nations,^12^^,^^32^, ^33^, ^34^, ^35^, ^36^, ^37^, ^38^, ^39^, ^40^, ^41^ we found Pacific EC and other frontline HCWs experienced physical exhaustion and mental health challenges exacerbated by workforce shortages, being stretched well-beyond capacity, limited access to mental health services, lack of appropriate compensation and incentive, and the experience of isolation, stigma and discrimination from colleagues, family and community. Participants spoke of the overlapping personal and professional tensions they faced in protecting their family and local community from COVID-19, versus their duty to serve their patients, profession, and country. This research also makes clear that many PICT EC providers and frontline HCWs do not feel recognised or valued for their role in service to their communities or colleagues. In some instances, their EC knowledge and training was not respected or recognised by national authorities and health and emergency systems leadership.

Lack of high-level recognition not only demoralises HCWs who are working enormous hours away from family and putting their lives on the line, but ultimately thwarts on-the-ground knowledge leadership that could help Pacific countries identify and implement localised, innovative, timely, culturally acceptable, and cost-effective pandemic interventions at the health service, system, and national policy levels. Proactive engagement of EC providers by Pacific leaders could positively offset the lack of strong health information systems and robust health data across the region. This lack of data impedes evidence-based health policy and planning not only during business-as-usual periods but dynamic public health emergencies and other disaster events common to the PICTs.^42^, ^43^, ^44^, ^45^ Thus by engaging EC and other frontline HCWs, country leaders can obtain authoritative, real-time information and guidance from reliable ‘ground zero’ sources.

Importantly, this study contributes to the limited qualitative studies describing healthcare professionals’ experiences during the COVID-19 pandemic worldwide. Longitudinal qualitative research expanding this study in the region is urgently needed. We recommend further investigation into HCW gendered experiences,^46^^,^^47^ use of lay personnel or community volunteers by EC providers to fight the pandemic, HCW interface with health information systems (notably medical certification of cause of death reporting in the context of countries’ civil registration and vital statistics (CRVS) systems for emergency public health policy and planning^48^), as well as HCW use of telehealth services, social media and other digital and communication channels and platforms. We also call for exploration of how local EC personnel collaborate with other human resources to manage the triple and sometimes quadruple threat of infectious disease outbreaks that constitute public health emergencies, *and* natural disasters (i.e., cyclones, sea-level rise and flooding), *and* emerging low or high-intensity conflicts (e.g., conflict flare-up in the Solomon Islands in November 2021), *and* the chronic disease ‘double pandemic’ in the Pacific region.^49^^,^^50^

This study has several limitations. The geographic region in focus has varied, ongoing experiences of COVID-19, as well as diverse histories, peoples, cultures, political and power structures, and socio-economic landscapes at national and subnational levels. As there are few HCWs and even fewer personnel with specialist EC skills and knowledge, it was important to ensure participant de-identification and confidentiality in reporting our findings. Thus, we were unable to disclose participant experiences vis-à-vis specific locations. For example, the differences in HCW experience within and among the three Pacific regions (Polynesia, Melanesia and Micronesia) could not be unpacked.

It is also worth noting three points considered by the research team around study framing. First, the complex challenges already facing the chronically understaffed EC and broader health workforce in the region was known at study commencement in March 2020.^2^^,^^3^^,^^31^ Therefore, the research team did not want a deficit approach taken that grounded study investigation in the pre-existing insufficient HCW numbers and poor patient-doctor ratios. Rather, we aimed to illuminate the voices, competency, ingenuity, responsiveness, and productivity of the highly committed HCWs at the fore of the Pacific region's COVID-19 pandemic response.

Second, study framing could suggest that when the WHO Director-General declared 2019-nCoV (COVID-19) a Public Health Emergency of International Concern on 30 January 2020, EC specialists and HCWs in the Pacific region (particularly in the Western Pacific Island region) were not already struggling to manage an active public health emergency, the non-communicable disease (NCD) crisis.^51^ Thus, when the COVID-19 pandemic spread in and to all global regions in 2020, it is erroneous to suggest many HCWs in our study were operating in a pandemic-free context. When COVID-19 hit they would be forced to face the double burden of the COVID-19 and NCD (or “twin”) pandemics.^52^^,^^53^

At study outset the third point of issue the research team recognised is that LMICs in the distinct Pacific region will have (and continue to have) very different, localised experiences of COVID-19 outbreak, transmission and response – and, at study mid-point, vaccination rates.^54^ In 2020, many PICTS leveraged their isolation to remain completely COVID-19 free, but in the first half of 2021 cases began to particularly surge in Papua New Guinea and Fiji, alarming communities and HCWs in neighbouring countries. By mid-2021, the first domestic transmission of the COVID-19 Delta variant was detected in Timor-Leste in August 2021 and in Vanuatu in late 2021.^55^^,^^56^ Consequently, we acknowledge that the experience of HCWs is not homogenous across the region.

Finally, it is important to note that throughout the data collection rounds in 2020 and early 2021, study participants frequently highlighted the significance of this research in making visible their collective professional and personal experiences for advancing impactful, transparent, accountable, and game-changing pandemic policy, planning and investment. They also emphasised the importance of the SPC and ACEM tele-mentoring and support sessions for contributing to their mental health, wellbeing and resilience – the data from which was foundational for this study (Box 3). Here, participant experience reinforces the benefits of anchoring pandemic HCW tele-mentoring and similar support activities in qualitative research for simultaneous, cost-effective South to South learning, bi-directional communication across the region and essential resource development for EC.

Box 3 **Example of participant views on the importance of remote pandemic HCW support**

*“*These ZOOM calls have been a strength… [They've] fast-tracked our communication with regional and international colleagues and potential partners that we'd otherwise simply not have had”

“It's business as usual here. Nobody, I think it's only the [SPC and] ACEM team, has come and pat us on the back and said thank you”

“This peer support has really [been] a strength… Just speaking to each other, and hearing each other, that provided a lot of comfort and support to our doctors and health workers on the ground… [relieving] a lot of anxiety”

Alt-text: Unlabelled box

## Conclusion

Recommendations arising out of this research align strongly with consensus priorities and standards that were identified by health stakeholders across the Pacific to develop and support EC systems prior to the outbreak of the COVID-19 pandemic.^3^ Urgent national, regional, and international investment and incentive must be given to the current and next generation of locally trained HCWs who are watching and wondering - why stay? There is evidence that the psychological distress from working during an infectious disease outbreak can persist for 2 to 3 years after the outbreak.^57^, ^58^, ^59^ As participants highlighted, EC is an integral part of UHC in the Pacific region and must be at the core of multi-sectoral health, sustainable development, and disaster policy, planning and investment.^60^ However limited, there is no point having health infrastructure, supplies and equipment if there is no one skilled (or too exhausted and demoralised) to use it.^61^^,^^62^ With such limited numbers of HCWs available for many Pacific nations, it is imperative that the dignity and welfare of local HCWs is prioritised and protected as essential resources not readily replaced. We encourage national and international actors to leverage study findings to prioritise development of robust and sustainable EC systems in the Pacific region, and globally.

## Contributors

MC, GP, RM, CEB, GOR and SK were primarily responsible for study design. DS, MK, PP and BK provided regional perspectives and contextual advice throughout all aspects of the project. All authors participated in data collection through online support forums, interviews or focus group discussions. LH and SK were responsible for transcription, with SK undertaking preliminary coding and presentation of data to the broader research team. CEB contributed to thematic analysis and secondary coding. MC, GP, RM, GOR, DS and LH contributed to data synthesis, including data triangulation, amalgamation and integration. CEB and SK developed the first draft of this manuscript. The final version was reviewed and approved by all authors.

## Data sharing statement

Study protocols and de-identified data that underpin these findings may be available (between 9 and 24 months after publication) to investigators whose proposed use of these data has been approved by an independent review committee, and subject to any restrictions imposed by relevant ethics committees, funders, the Australasian College for Emergency Medicine or the Pacific Community. Proposals to use the data may be submitted, and data made available, without investigator support.

## Declaration of interests

MC, GP, RM and GOR declare they are recipients of International Development Fund Grants from the Australasian College for Emergency Medicine Foundation. GP reports past research funding from the Pacific Community (SPC) and visiting Faculty status at the University of Papua New Guinea and Fiji National University. Additionally, RM reports grants from the Australian Government Department of Foreign Affairs and Trade as well as scholarships from the National Health and Medical Research Council (NHMRC) and Monash University. GOR reports that he is the recipient of an NHMRC Early Career Research Fellowship. CEB reports past research consultancy funding from SPC.
